# Molecular basis for the DNA damage induction and anticancer activity of asymmetrically substituted anthrapyridazone PDZ-7

**DOI:** 10.18632/oncotarget.21806

**Published:** 2017-10-10

**Authors:** Majus Misiak, Mateusz Heldt, Marlena Szeligowska, Stefania Mazzini, Leonardo Scaglioni, Grzegorz J. Grabe, Marcin Serocki, Jan Lica, Marta Switalska, Joanna Wietrzyk, Giovanni L. Beretta, Paola Perego, Dominik Zietkowski, Maciej Baginski, Edward Borowski, Andrzej Skladanowski

**Affiliations:** ^1^ Department of Pharmaceutical Technology and Biochemistry, Faculty of Chemistry, Gdansk University of Technology, Gdansk, Poland; ^2^ Department of Food, Environmental and Nutritional Sciences, Division of Chemistry and Molecular Biology, University of Milan, Milan, Italy; ^3^ Department of Medicine, Faculty of Medicine, Imperial College London, London, UK; ^4^ Molecular Pharmacology Unit, Department of Experimental Oncology and Molecular Medicine, Fondazione IRCCS Istituto Nazionale dei Tumori, Milan, Italy; ^5^ Department of Experimental Oncology, Hirszfeld Institute of Immunology and Experimental Therapy, Polish Academy of Sciences, Wroclaw, Poland; ^6^ BS-154 sp. z o.o., Gdansk, Poland

**Keywords:** actin, anthraquinone, cell cycle, DNA repair, topoisomerase

## Abstract

Anthrapyridazones, imino analogues of anthraquinone, constitute a family of compounds with remarkable anti-cancer activity. To date, over 20 derivatives were studied, of which most displayed nanomolar cytotoxicity towards broad spectrum of cancer cells, including breast, prostate and leukemic ones. BS-154, the most potent derivative, had IC_50_ values close to 1 nM, however, it was toxic in animal studies. Here, we characterize another anthrapyridazone, PDZ-7, which retains high cytotoxicity while being well tolerated in mice. PDZ-7 is also active *in vivo* against anthracycline-resistant tumor in a mouse xenograft model and induces DNA damage in proliferating cells, preferentially targeting cells in S and G_2_ phases of the cell cycle. Activation of Mre11-Rad50-Nbs1 (MRN) complex and phosphorylation of H2AX suggest double-stranded DNA breaks as a major consequence of PDZ-7 treatment. Consistent with this, PDZ-7 treatment blocked DNA synthesis and resulted in cell cycle arrest in late S and G_2_ phases. Analysis of topoisomerase IIα activity and isolation of the stabilized covalent topoisomerase IIα - DNA complex in the presence of PDZ-7 suggests that this compound is a topoisomerase IIα poison. Moreover, PDZ-7 interfered with actin polymerization, thereby implying its action as a dual inhibitor of processes critical for dividing cells. Using nuclear magnetic resonance (NMR) spectroscopy we show that PDZ-7 interacts with DNA double helix and quadruplex DNA structure. Taken together, our results suggest that PDZ-7 is a unique compound targeting actin cytoskeleton and DNA.

## INTRODUCTION

Analogues of anthraquinone, including anthracyclines (doxorubicin and its derivatives) and mitoxantrone, constitute a class of highly active anti-cancer drugs used broadly in the treatment of wide range of malignancies, from leukemias and lymphomas to solid tumors [1]. Anthracyclines are frequently assigned, along with structurally-unrelated drugs amsacrine and etoposide, to a broader class of type II DNA topoisomerase inhibitors [2, 3]. In vertebrates, two isoforms of type II topoisomerase are present: topoisomerase IIα (Topo IIα) and IIβ (Topo IIβ). These enzymes regulate DNA structure by creating transient double strand break in one DNA helix and passing another helix through the gap, before its resealing [3]. Topo IIα is indispensable for proliferating cells [4] where it is expressed at high levels. Topo IIα is required for DNA replication, condensation of mitotic chromosomes and, to lesser extent, transcription [5], whereas Topo IIβ is partially redundant and is also expressed in non-proliferating cells. Drugs interfering with topoisomerase catalytic cycle at the helix resealing step (referred to as topoisomerase poisons) lead to formation enzyme-associated DNA single- and double-strand breaks and are in consequence strongly cytotoxic [2, 3]. DNA interacting agents including Top2 inhibitors trigger a DNA damage response (DDR), which involves multiple proteins acting in sensing and repairing the damage [6]. Specifically, DNA lesions activate a network of signaling pathways which delay cell cycle progression and activate repair mechanisms. Multiple phosphorylation events are triggered in the DDR response, many of which are carried out by ATM, acting in concert with the Mre11-Rad50-Nbs1 (MRN) complex [7]. Though some topoisomerase II poisons, like etoposide, are extremely specific [8], anthraquinone analogues can exert their activity also by DNA-crosslinking and alterations in chromatin structure [9–11].

Administration of chemotherapy regimens based on anthracyclines is limited by accumulative cardiotoxicity resulting from reactive oxygen species (ROS) generated during iron-dependent redox cycling of the anthraquinone moiety [12]. Off-target poisoning of Topo IIβ by the Topo IIα inhibitors is thought to be the underlying cause of secondary leukemias induced by chemotherapy [13, 14] and has been argued to contribute to cardiotoxicity [15]. Even with the use of complex liposomal formulations the cardiotoxicity remains a major limitation of anthracyclines in the clinic [16]. The toxicity of currently used regimens could be partially solved by replacing anthracyclines with structural analogues selectively targeting Topo IIα and not generating ROS [17, 18]. This approach was found to be effective in the case of imidazoacridones, a group of type II topoisomerase inhibitors [19]. One compound from this family, C-1311, entered clinical trials and proved to be both well-tolerated in humans and non-cardiotoxic [20].

**Figure 1:**
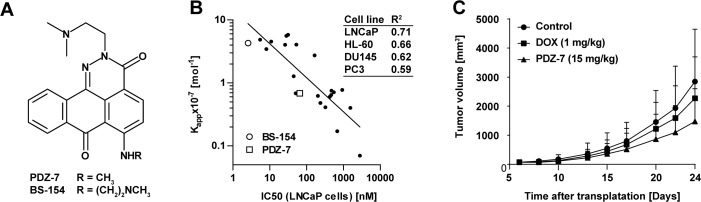
PDZ-7 is a DNA binding compound with promising *in vivo* properties **(A)** Chemical structures of anthrapyridazones PDZ-7 and BS-154. **(B)** Tumor growth inhibition by PDZ-7. Athymic Foxn1nu mice were transplanted subcutaneously with doxorubicin-resistant LoVo/DX colon cancer cells, and xenografts were allowed to grow to 70 mm^3^. Mice were injected with PDZ-7 (15 mg/kg) or doxorubicin (ADR, 1 mg/kg) on days 6, 13 and 20 following tumor transplantation. Mean tumor volumes [mm^3^] are plotted against days after transplantation for control and drug treated mice (10 mice were used for each condition). Both PDZ-7 and doxorubicin were used in comparable dose of c.a. 0.3 maximum tolerated dose (MTD). **(C)** Comparison of DNA affinity of 23 anthrapyridazones, defined as apparent DNA binding constant (K_app_, 10^−7^ Mol^−1^) and cytotoxicity in 4 cell models: LNCaP, PC3, DU145 (prostate cancer) and HL-60 (acute myeloid leukemia). Linear determination coefficients (R^2^) calculated for each pair of values are shown in the inset. Two selected compounds, BS-154 and PDZ-7 are explicitly marked. DNA affinity was measured by ethidium bromide displacement assay and cytotoxicity by manual counting after 72-hour exposure to drugs. Original data are disclosed in the granted US Patent [11].

Anthrapyridazones (2,7-dihydro-3*H*-dibenzo[*de, h*]cinnoline-3,7-diones) constitute a family of imino- analogues of anthraquinone with potent activity [21]. Symmetric derivatives displayed cytotoxicity in nanomolar range and were active towards doxorubicin-resistant leukemia cells [21] among others. Asymmetrically substituted anthrapyridazones were cytotoxic in low nanomolar concentrations against prostate, breast and colon cancer cells [22] and retained activity against multi-drug resistant cells overexpressing ABCB1 and ABCC1 membrane transporters. Like other anthraquinones, anthrapyridazones strongly bind to DNA, but the exact mechanism of their pharmacological action is unclear. In this report, we describe the molecular mechanism of action of compound PDZ-7 (Figure 1A), an anthrapyridazone with promising preclinical properties. We compare PDZ-7 with BS-154, the most potent anthrapyridazone derivative characterized to date, in the context of DNA damage induction.

## RESULTS

### PDZ-7 is a potent anticancer compound and an inhibitor of DNA topoisomerase IIα

As BS-154 compound had a potent cytotoxic activity in preclinical models including cell lines derived from various tissues, we tested whether this compound exhibits similar anti-cancer activity *in vivo*. When Administered intravenously 3 times once a week to BALB/c mice, BS-154 was toxic at doses of 0.75 to 1 mg/kg (results not shown), suggesting a too narrow therapeutic window for a prospective anti-cancer drug (MTD<0.75 mg/kg). Conversely, compound PDZ-7 was well tolerated by mice at doses up to 50 mg/kg (single dose), causing transient decrease in mice body weight at the highest dose (Table 1) and no anatomopathological abnormalities (not shown). For the preliminary studies of *in vivo* anti-cancer activity LoVo/DX (human colon cancer cells with multidrug resistance (MDR) phenotype [23]) xenograft mice model was selected. LoVo/DX cells were subcutaneously transplanted into Foxn1nu mice and measured tumor growth inhibition relative to untreated mice over 8 to 24 days. Mice were treated with three doses once a week (on days 6, 13, 20) of 15 mg/kg PDZ-7 or 1 mg/kg doxorubicin. The doses were adjusted for the relative toxicity of each drug, with each one amounting to *ca.* 0.3 MTD. Doxorubicin treatment resulted in 15-28% tumor growth inhibition, whereas treatment with PDZ-7 inhibited tumor growth by 36-48% when compared to untreated mice (Figure 1B). These results demonstrated that PDZ-7 is well tolerated in mice and has anti-cancer activity *in vivo* significantly better than doxorubicin at comparable doses in this model. It encouraged us to carry out studies to determine its mechanism of action.

**Table 1:** Effect of PDZ-7 administration on BALB/c mice body weight

PDZ-7	Body mass [g], days after injection				
	1	3	5	8	10
10 mg/kg	21.2 ± 0.3	21.2 ± 0.7	21.2 ± 0.7	21.5 ± 1.0	21.6 ± 0.8
15 mg/kg	21.5 ± 1.0	20.6 ± 1.2	20.6 ± 1.0	20.6 ± 0.9	20.9 ± 1.5
30 mg/kg	20.6 ± 1.6	20.4 ± 1.7	20.2 ± 1.8	20.7 ± 2.0	21.7 ± 1.7
50 mg/kg	20.8 ± 0.6	20.5 ±1.0	18.1 ± 0.9	19.9 ± 1.2	20.5 ± 1.0

BALB/c mice were injected with a single dose of PDZ-7 (10, 15, 30 or 50 mg/kg) in the tail vein and body weight was measured every day for a period of 2 weeks. Mean mice weight ± SD for 3 mice is shown for selected time points. No statistically relevant changes were noticed.

**Figure 2:**
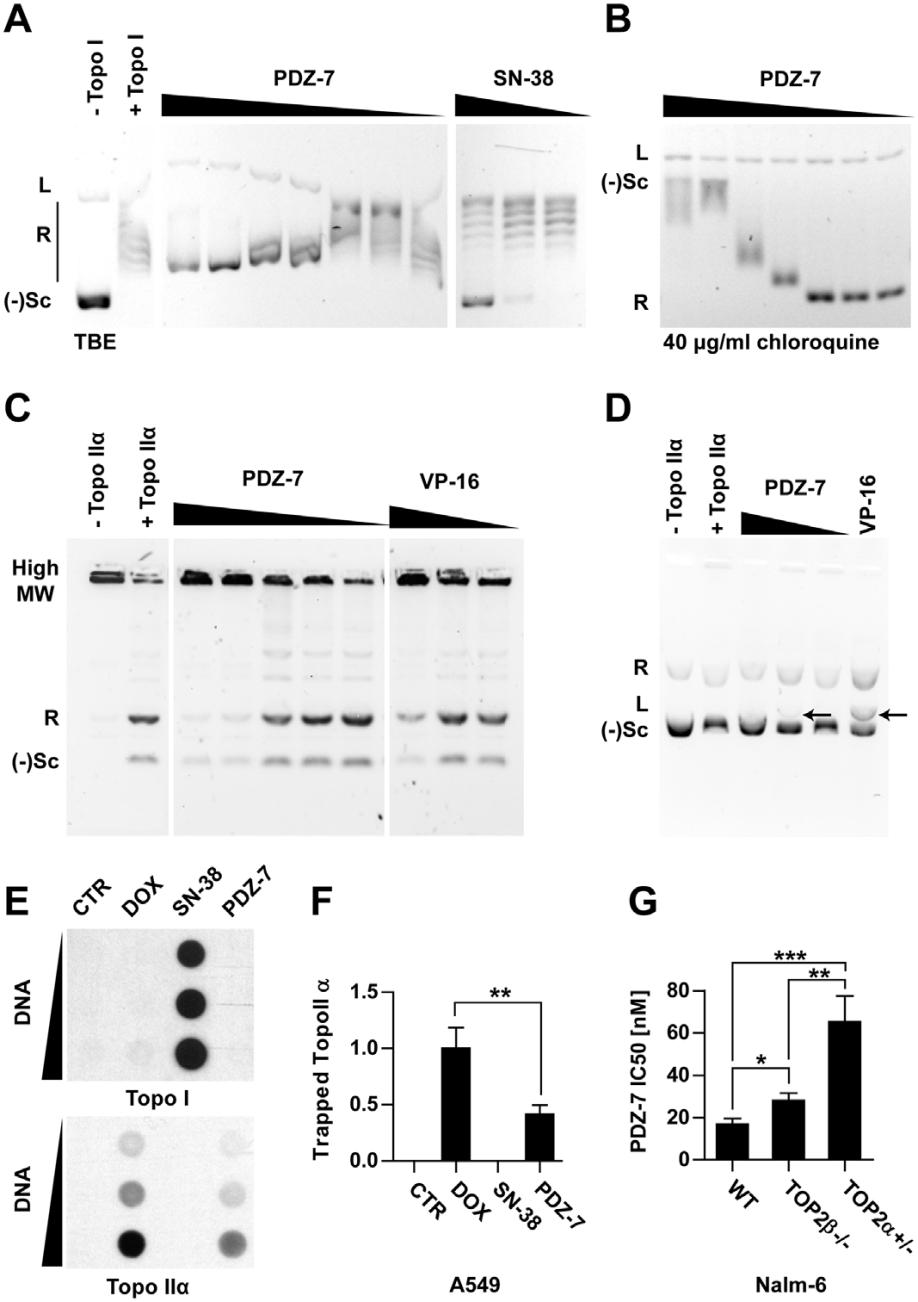
Anthrapyridazone PDZ-7 is topoisomerase IIα inhibitor **(A)** Inhibition of topoisomerase I-mediated pBR322 relaxation. Reaction was performed either with topoisomerase I alone (+ Topo I), or in the presence of drugs: PDZ-7 (0.1, 0.5, 1, 5, 10, 50 or 100 μM) and SN-38 (0.1, 1 or 10 μM). Bands corresponding to linear (L), relaxed (R) and negatively supercoiled ((−)Sc) forms of pBR322 are marked. **(B)** Same PDZ-7 samples run in the presence of 40 μg/ml chloroquine, a DNA intercalator which causes inversion of R and (−)Sc bands and displaces PDZ-7 from DNA. **(C)** Inhibition of topoisomerase IIα mediated kDNA decatenation by PDZ-7 (0.1, 0.5, 1, 5 and 10 μM) in comparison with etoposide (1, 10 and 100 μM). **(D)** pBR322 cleavage by topoisomerase IIα in the presence of PDZ-7 (0.1, 1, 10 μM) or etoposide (100 μM). Cleavage product (L, linear DNA) is marked with arrows. **(E)** Detection of covalent topoisomerase-DNA complexes (ICE assay) in A549 cells treated for one hour with DMSO (CTR), 10 μM doxorubicin (ADR), SN-38 or PDZ-7. **(F)** Densitometric quantification of data shown in E (mean ± S.D.), calculated from 4 independent experiments. **(G)** PDZ-7 sensitivity of Nalm-6 cells with homozygous deletion of topoisomerase IIβ (TOP2β−/−) or heterozygous deletion of topoisomerase IIα (TOP2α+/−) in comparison with wild-type (WT) cells. Data is shown as IC50 calculated from 3 independent experiments. Statistical significance of data in F and G was determined with two-tailed Student t-test.

Using publicly available data [22] correlation plots between apparent DNA binding constants (K_app_) and 72 hour cytotoxicity for 23 anthrapyridazone derivatives (Figure 1C) were generated. Depending on the cell line type, linear determination coefficients (R^2^) ranged from 0.59 to 0.71, pointing to DNA as the site of action for these drugs. Since our correlation data indicated that PDZ-7 can act as a DNA binding agent [22] it was tested if PDZ-7 can affect DNA topoisomerases or their DNA-complexed forms. Firstly, PDZ-7 was tested in a topoisomerase I-mediated pBR322 relaxation assay. Addition of PDZ-7 inhibited plasmid relaxation and led to a band shift of negatively supercoiled plasmid (Figure 2A). This result suggested that PDZ-7 could bind DNA. We then repeated the electrophoresis in the presence of a DNA intercalating agent (chloroquine) in an attempt to displace PDZ-7 bound to DNA. Chloroquine causes relaxation of negatively supercoiled plasmid and transition of relaxed plasmid to supercoiled state, effectively leading to reversal of supercoiled and relaxed bands, and allows to separate nicked circular DNA from the supercoiled form [24]. Under these conditions band migration normalized and PDZ-7 was found to completely inhibit Topo I at concentrations of 50 and 100 μM (Figure 2B). An increased DNA cleavage in the entire concentration range of 0.1-100 μM (Figure 2B) was not observed.

Secondly, the experiment with topoisomerase IIα (Topo IIα) was conducted and resulted in observation that PDZ-7 also inhibits Topo IIα *in vitro*, fully preventing kinetoplast DNA (kDNA) decatenation at concentrations of 5 μM or higher (Figure 2C). However, PDZ-7 did not induce DNA cleavage as efficiently as etoposide (VP-16), a non-intercalative topoisomerase poison [25] (Figure 2D). A faint band corresponding to linear pBR322 plasmid was observed only at 1 μM and not at higher or lower concentrations of PDZ-7 (Figure 2D). This suggests that in case of PDZ-7 mediated plasmid cleavage the dose-response follows a bell-shaped curve.

To assure that the result of *in vitro* topoisomerase inhibition is relevant for drug cytotoxic activity, A549 cells were exposed to equal (10 μM) concentrations of PDZ-7, SN-38 and doxorubicin (DOX). High concentrations, in comparison to subsequent studies, were chosen to compensate low limit of detection of the ICE assay. Following one hour treatment, covalent DNA-protein complexes were isolated by ultracentrifugation (Figure 2E). Interestingly, it was evidenced that PDZ-7 specifically induced protein-DNA complexes only in the case of Topo IIα, but not Topo I (Figure 2E). When compared with doxorubicin, the amount of Topo IIα-DNA complex isolated from cells treated with PDZ-7 was over 2 times lower (Figure 2F). Thus PDZ-7 is a strong inhibitor of topoisomerases I and II *in vitro*, likely due to its high DNA affinity, however this compound induces relatively low level of covalent Topo IIα-DNA complexes. Topo IIα as a target for PDZ-7 was further confirmed by comparing its activity towards cells with altered expression of topoisomerase forms. Nalm-6 pre-B leukemia cells with heterozygous deletion of Topo IIα (Nalm-6^TOP2α+/−^) or homozygous deletion of Topo IIβ (Nalm-6^TOP2β−/−^) [17, 26] were 4.0 times and 1.5-time more resistant to PDZ-7, respectively (Figure 2G). Altogether these results demonstrate that PDZ-7 can cause DNA damage by poisoning both type II topoisomerases. However, higher sensitivity of Nalm-6^TOP2α+/−^ cells in comparison to wild-type and Nalm-6^TOP2β−/−^ suggests that Topo IIα is a preferred target of PDZ-7 in cells.

### PDZ-7 and BS-154 are potent inducers of DNA damage

**Figure 3:**
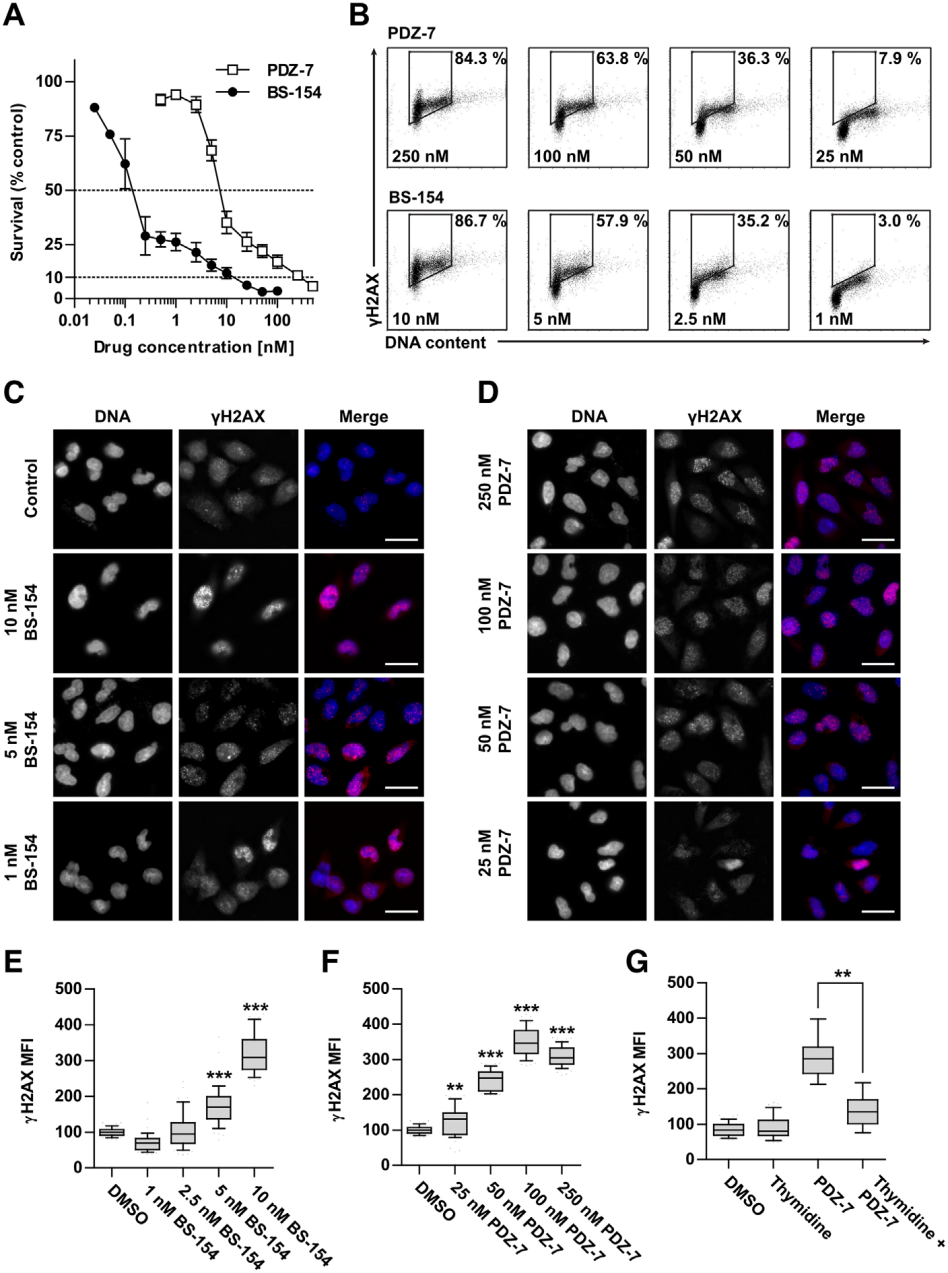
Anthrapyridazones BS-154 and PDZ-7 induce cytotoxic double stranded DNA breaks **(A)** Cytotoxicity of PDZ-7 and BS-154 towards A549 cells after 120 hours of continuous exposure to each drug, determined by MTT assay. Each point represents mean ±S.D. from three independent experiments. **(B)** Induction of DNA strand breaks after 1 hour of drug treatment with regard to cell cycle phase. A549 cells were stained with propidium iodide (DNA content) and anti-γH2AX antibody conjugated to Alexa-594. Percentage of cells with high γH2AX level is shown for each scatter plot. (**C** and **D**). Dose dependent formation of γH2AX *foci* after 1 hour of treatment with PDZ-7 and BS-154 shown by immunofluorescence. DNA was counterstained with DAPI. Scale bar: 25 μm. (**E** and **F**) quantification of the median γH2AX fluorescence signal intensity over the nucleus area in. **(G)** Asynchronously growing or thymidine synchronized A549 cells were treated with 250 nM PDZ-7 for 1 hour, before anti-γH2AX staining. Quantification of γH2AX was performed as in E and F. Each box in panels E-G represents data for 50 nuclei scored and statistical significance was determined using two-tailed Mann-Whitney test.

Since A549 Non-Small Cell Lung Cancer cell line is extensively studied as a model for DNA damaging drugs [27–31], it was selected to further elucidate the mechanisms of action of the studied compounds. Both PDZ-7 and BS-154 displayed potent cytotoxicity towards A549 cells, with their respective IC50 values of 6.8 ± 1.5 nM and 0.14 ± 0.4 nM (Figure 3A). Growth inhibition curves were biphasic, with IC50 and IC90 values spread far apart (Figure 3A). The latter was approximately 250 nM for PDZ-7 and 10 nM for BS-154 (Figure 3A). IC90 concentration of compound PDZ-7 was selected as the main reference for further studies on mechanism of action.

It was also investigated whether the differences in PDZ-7 and BS-154 activity result from differential ability to cause direct or enzyme-mediated DNA strand breaks in cells. Using p-Ser^139^-H2AX (γH2AX) as a marker of DNA breaks [32] γH2AX *foci* through immunofluorescence and flow cytometry in cells exposed to drugs were detected (Figure 3B-3D). Additionally, a uniform level of γH2AX phosphorylation throughout S and G_2_ phases was observed (Figure 3B) and markedly lower staining of early G_1_ cells, independent of the drug or concentration used. This finding indicated that DNA damage was preferably induced in proliferating cells. One hour exposure of A549 cells to PDZ-7 and BS-154 at concentrations corresponding to their respective IC70 – IC90 doses resulted in a concentration-dependent increase in histone H2AX phosphorylation and over 3-fold increase above the background level, attributed to endogenous DNA damage [33] (Figure 3E and 3F). Interestingly, γH2AX *foci* changed in morphology following increasing concentrations: at IC70 (25 nM of PDZ-7 and 1 nM of BS-154) a small and diffused *foci* were observed and gradually coalesced to grainy staining at IC90 concentrations, likely resulting from double-strand DNA break (DSB) induction (Figure 3C and 3D). The signal intensity of γH2AX for PDZ-7 and BS-154 demonstrated a dose-response relationship (Figure 3C-3F). BS-154 was stronger in *foci* induction, by over an order of magnitude, yet equitoxic concentrations of both drugs induced comparable levels of DNA damage, suggesting that their cytotoxicity is a direct consequence of DNA breaks. Synchronization of A549 cells in G_1_ phase by thymidine block reduced (2-fold) PDZ-7 activity consistently with its specificity towards S/G_2_ cells (Figure 3G).

**Figure 4:**
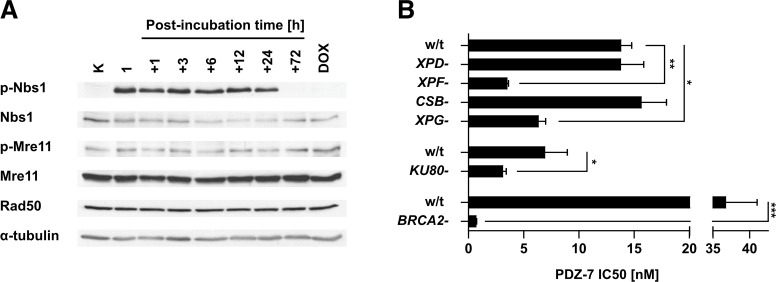
PDZ-7 induces DNA damage response and is selectively cytotoxic towards DNA repair-deficient cells **(A)** Induction of DNA-damage response over the course of PDZ-7 treatment. A549 cells were treated with 250 nM PDZ-7 for 1 hour and post-incubated in drug-free medium up to 72 hours. Activation of MRN complex was determined by Western Blotting, using antibodies towards Nbs1 phosphorylated at Ser^343^ (p-Nbs1), Mre11 at Ser^676^ (p-Mre11) and total Rad50. **(B)** CHO AA8-derived UV sensitive mutants with defects in nucleotide excision repair pathway displayed specific sensitivity to PDZ-7. Nuclease-deficient variants UV41 (*XPF-*) and UV135 (*XPG-*) were 4.0 and 2.2 times more sensitive (IC_50_ value), compared to cells with defects in damage recognition (UV61, *CSB-*) and in helicase activity (UV5, *XPD-*). Loss of Ku80 protein involved in NHEJ repair in xrs6 cells amounted to 2.2-fold increase in sensitivity in comparison to parent CHO-K1 cells. V-C8 cells deficient in DNA damage repair via HR (*BRCA2-*) had 53-fold lower PDZ 7 IC50 value in comparison to parent V79 cells. Statistical significance was determined using two-tailed Student t-test.

To confirm that the grainy γH2AX staining results from DSB, activation of Mre11-Rad50-Nbs1 (MRN) DSB repair complex [34] in PDZ-7 treated cells was measured. After 1-hour exposure to 250 nM PDZ-7 followed by post-incubation in drug-free medium an increased phosphorylation of Nbs1 at Ser^343^ that lasted for 24 hours was observed (Figure 4A). Elevated phosphorylation of Nbs1 points to induction of DSB by PDZ-7 and activation of DNA damage response. Then it was further tested whether PDZ-7 induces DNA damage other than DSB. For this purpose, a panel of hamster cell lines with deficient DNA repair pathways was used, including UV-hypersensitive mutants deficient in nucleotide-excision repair (NER), a DNA repair system responsible for the removal of DNA adducts [35]. A sensitivity of cells deficient in non-homologous end-joining (NHEJ) and homologous recombination (HR) DSB repair pathways (Figure 4B) was also compared. Cells deficient in endonucleases known to excise damaged DNA fragments, *XPF* and *XPG*, were more sensitive to PDZ-7 (Figure 4B). On the contrary, *CSB*- and *XPD*- CHO cells deficient in damage recognition and helicase activity, respectively, were equally sensitive to PDZ-7 (Figure 4B). A higher sensitivity of cells deficient in two pathways of DSB repair – xrs6 lacking *KU80* (NHEJ pathway) and V-C8 cells lacking *BRCA2* (HR pathway) (Figure 4B) were also observed. Hypersensitivity of *BRCA2*-deficient cells to other reports [36, 37] and highlights the role of DSB formation in PDZ-7 cytotoxicity.

### PDZ-7 induces abnormal perturbations in the cell cycle

**Figure 5:**
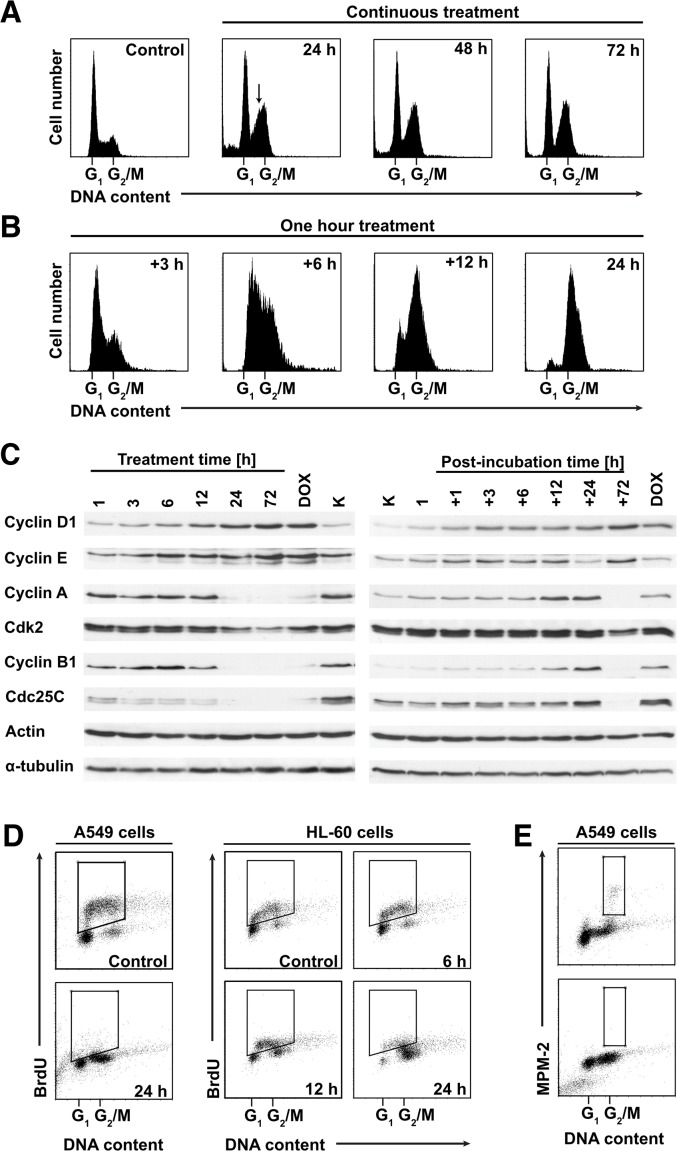
PDZ-7 induces unusual perturbations in cell cycle **(A)** Continuous exposure to PDZ-7 led to atypical dual G_1_ / late S cell cycle arrest, which persisted up to 72 hours. **(B)** Short treatment (one hour) and post-incubation in drug-free medium induced synchronous progression through S phase and G_2_ arrest. **(C)** Expression of cell cycle regulating proteins during prolonged and short treatment times was evaluated by Western Blotting. **(D)** Inhibition of DNA synthesis and S-phase progression by IC90 concentrations of PDZ-7 observed independently in two model cell lines: A549 and HL-60 (acute myeloid leukemia). DNA synthesis was determined by BrdU incorporation into nascent DNA and detection with anti-BrdU antibodies. BrdU-positive cells are shown for each scatter plot. **(E)** Inhibition of mitotic activity by PDZ-7, determined by staining for mitosis-specific MPM-2 phosphoepitope.

Continuous treatment with 250 nM PDZ-7 induced unusual arrest in G_1_ and late S phases of the cell cycle which lasted for 72 hours (Figure 5A). Concomitantly with G_1_ block, cells that were in early S phase at the time of treatment initiation progressed to late S, before arresting. This unusual behavior was associated with degradation of cyclin B1 and cdc25c and stabilization of both full-length and low molecular weight isoforms of cyclin E (Figure 5C). Brief exposure (1 hour PDZ-7 and post-incubation in drug-free medium) induced slow, synchronous progression through S phase until arrest in G_2_, accumulation of cyclins A and B1 (Figure 5B). Progression through S phase peaked between 6 and 12 hours, before cells finally stopped in late G_2_, as expected from high accumulation of cyclin B1 (Figure 5C). In fact, this kind of response during treatment is typical for DNA damaging drugs. Moreover, PDZ-7 treatment resulted in complete cessation of DNA synthesis, revealed by BrdU incorporation after 24 hours (Figure 5D) and arrest of mitotic activity, determined by MPM-2 staining (Figure 5E).

### Interaction of PDZ-7 with the cytoskeleton

**Figure 6:**
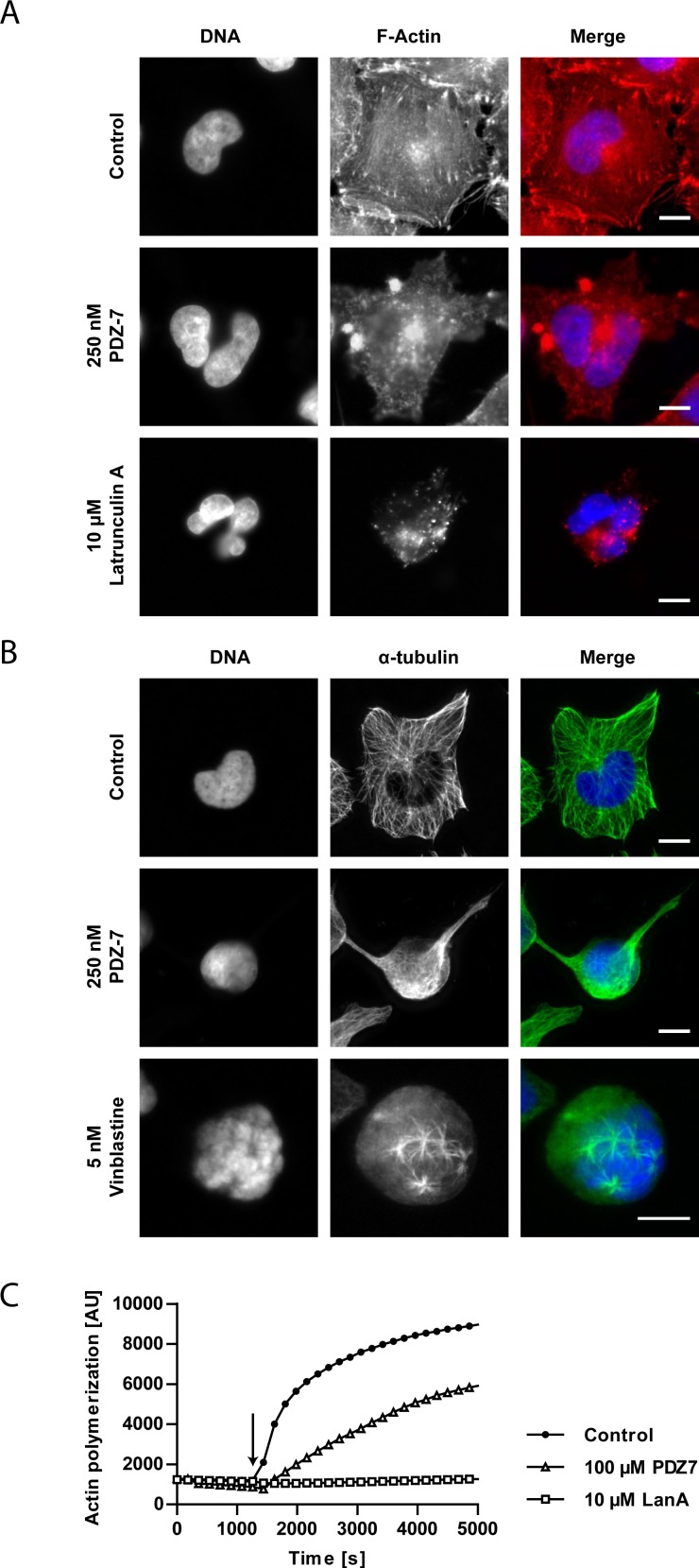
PDZ-7 causes cytoskeleton destabilization in A549 cells **(A)** Changes in phalloidin-stained F-actin cytoskeleton morphology and **(B)** changes in α-tubulin cytoskeleton in A549 cells treated with 250 nM PDZ-7 for 24 hours. 10 μM latrunculin A and 5 nM vinblastine were used as positive controls for A and B, respectively. Scale bar: 10 μm. **(C)** Polymerization of rabbit muscle G-actin to F-actin in the presence of ATP and DMSO (control) or studied drugs: 100 μM PDZ-7 or 10 μM latrunculin A (LanA). Arrow points to polymerization initiation. Note lower initial amount of F-actin in PDZ-7 sample, compared to control.

The abnormally rounded morphology of A549 cells exposed to PDZ-7 for longer periods (>12 hours) was observed. It was speculated that PDZ-7 could compromise cytoskeleton functions independently of DNA damage response induction. To verify this, the fluorescent co-staining of F-actin and α-tubulin in cells exposed to PDZ-7 was performed (Figure 6A and 6B). In cells exposed to PDZ-7 for 24 hours an actin aggregation in cortical region of the cell and disassembly of actin stress fibers, leading to cytokinesis failure and formation of bi-nucleated cells was observed (Figure 6A). α-Tubulin cytoskeleton collapsed along with actin depolymerization, leading to cell rounding. Despite that, polymerized microtubules were still present in PDZ 7 treated cells, in stark comparison to vinblastine treated cells (Figure 6B, middle and bottom panels). PDZ-7 did not cause α-tubulin depolymerization in treated cells, in contrast to actin. Moreover these cells were arrested in late S/G_2_ phases rather than during mitosis, as evidenced by (i) lack of staining for MPM-2, a mitotic phosphoepitope [38] (Figure 5E), (ii) no spindle formation and (iii) no chromatin condensation (Figure 6B). It was further supposed that morphology changes resulted from actin depolymerization rather than downregulation as neither total actin nor α-tubulin was altered in drug-treated cells (Figure 5C). To confirm that F-actin is a direct target for PDZ-7, an *in vitro* actin polymerization assay was performed. PDZ-7 inhibited G-actin polymerization and, in contrast to latrunculin A (LanA), it also caused depolymerization of residual F-actin present before the reaction was initialized (Figure 6C).

### DNA binding mode of PDZ-7

High propensity to induce DNA strand break and sensitivity of NER-deficient cells to PDZ-7 hinted that this compound may interact with DNA in unusual manner and perturb its structure, rather than simply inhibit topoisomerase IIα. To address this issue, the interaction of PDZ-7 with self-complementary double helix oligomers d(GCTACG)_2_ and d(AAGAATTCTT)_2_ used as models for CG- and AT-rich DNA sequences, respectively were studied.

**Figure 7:**
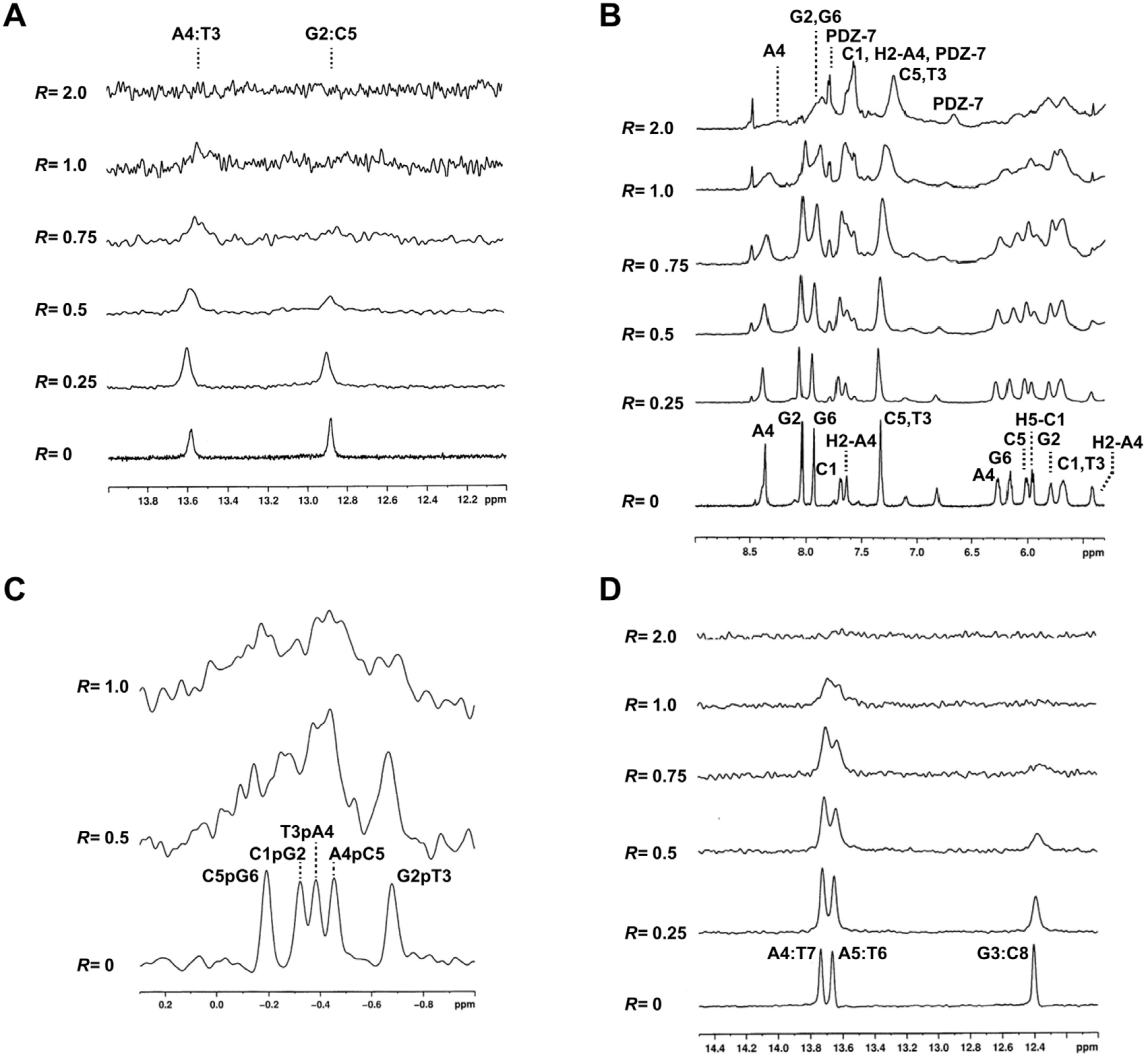
PDZ-7 does not bind DNA by intercalation **(A)** Imino proton region of d(CGATCG)_2_ duplex titrated with PDZ-7 from *R*= 0 to 2.0. **(B)** Aromatic and anomeric proton region of the same spectrum. **(C)** ^31^P spectrum of d(CGATCG)_2_ duplex titrated with PDZ-7. **(D)** Imino proton region of d(AAGAATTCTT)_2_ duplex titrated with PDZ-7 from *R*= 0 to 2.0.

^1^H and ^31^P NMR titration experiments were performed for both oligonucleotides to find possible contacts between PDZ-7 and nucleotides. Addition of a small amount of PDZ-7 (*R,* defined as drug to oligonucleotide ratio between 0.5 and 0.75), caused broadening of the signals of both the exchangeable (12-14 ppm) and the nonexchangeable protons without relevant chemical shift variations (Figure 7A, and 7D). In particular, the GC imino protons almost completely disappeared in the noise at *R*= 0.75. ^31^P NMR was further used, which is very sensitive to small changes in the geometry of the backbone caused by the interaction with the drugs. In our experience, intercalation process is associated with a lowfield shift up to 1.0–1.5 ppm because of the helix unwinding necessary for accommodation of the drug molecule, whereas an electrostatic association produces only small and generalized upfield shifts [39–44]. ^31^P NMR spectra of d(CGTACG)_2_, performed at *R=* 0.5 and 1 showed a notable line broadening without chemical shift variations (Figure 7C). This spectroscopic behavior can exclude an intercalation process and suggests a non-specific external interaction of the PDZ-7 with the double helix. To better investigate the chemical equilibria and to evaluate the relevant species present in solution, a DOSY experiment was performed on d(CGTACG)_2_/PDZ-7 complex. The diffusion coefficient value (D1) obtained is equivalent to a molecular weight of 7.7 kDa, which corresponds to one double helix associated with 10 PDZ-7 molecules or multimeric species in solution. This result is consistent with an aggregation process of the PDZ-7, likely due to the stacking of the aromatic moiety. The significant broadening of all the signals, even for *R*= 2, did not allow to detect NOE contacts between PDZ-7 and both oligonucleotides. Therefore, the interaction of PDZ-7 with oligonucleotides was not intercalative but rather occurred externally between the drug and negatively charged backbone of the double helix. Moreover, broadening of both AT and GC imino protons, which became generalized at higher drug to DNA ratios, suggested low specificity of the binding.

**Figure 8:**
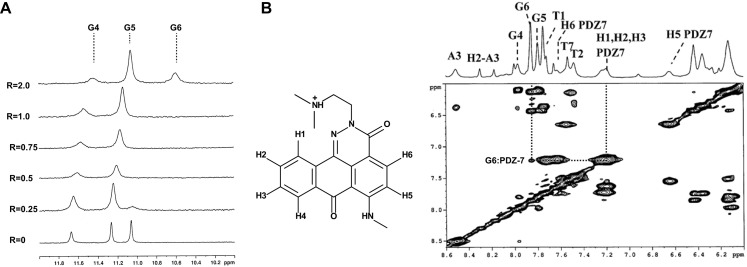
PDZ-7 binds duplex and quadruplex (G4) DNA **(A)** Imino proton region of the ^1^H NMR titration spectra of duplex oligonucleotide d(TTAGGGT)_4_ with PDZ-7 at 25°C. **(B)** Proton assignment for PDZ-7 and NOESY spectrum of PDZ-7-d(TTAGGGT)_4_ complex at *R* = 3.S.

Furthermore, it was interesting to check if PDZ-7 can interact more specifically with G-quadruplex DNA structures, presumably formed in telomeric and other G-rich DNA regions of chromosomes [45]. We used d(TTAGGGT)_4_ as a model of *in vitro* G-quadruplex-forming oligonucleotide that does not aggregate and is known to form a four stranded G–quadruplex complex (parallel strand orientation) [46]. During the titration of PDZ-7 in d(TTAGGGT)_4_ solution the original signals of G4-G6 imino experiment exhibited an upfield shifts (Table 2) broadened at high *R* values. G4NH changed its shape together with the G6 NH imino proton and disappeared at low *R*, but sharpened again for *R*= 2.0 (Figure 8A). The signals of the ligand in the complex (Figure 8B) were broad as well, likely due to the movement of the PDZ-7 inside the binding site and as a consequence of equilibrium between the free and the bound ligand (Table 3). Again, line broadening and the overlapping of aromatic protons of the oligonucleotide with PDZ-7 aromatic protons made it difficult to observe NOE signals. Nonetheless, few NOEs involving the G6 unit (H8 and H2′-H2′’) were detected with H2-H3 aromatic protons of the PDZ-7 (Figure 8B), together with the broadening and upfield shift of G6 NH (−0.5 ppm), suggesting that one binding site at the level of G6 is likely to be involved. Drug association with G4 did not alter composition of G tetrad, as follows from the characteristic inter-strand NH-NH interactions (not shown). Interestingly, it was observed that the A-tetrad was not conserved, evidenced by the lack of the NOEs between G4NH with A3NH_2_ and A3H2, which are typical interactions of the A tetrad, as well as the NOE of A3H8 with A3H2. Thus it is conceivable that a second binding site could be at the level of G4 residue even if no NOEs interactions between PDZ-7 and G4 were found. Interaction of PDZ-7 at the level of G6 tetrad suggests that it can form only a cap-complex with G-quadruplex.

**Table 2:** Chemical shift values of the complex PDZ-7/d(TTAGGGT)_4_

	H2/NH/Me		H6/H8		H1′		*H2′/H2”*	
	*δ*	*Δδ^b^*	*δ*	*Δδ*	*δ*	*Δδ*	*δ*	*Δδ*
T1	N.D.	N.D.	7.74	**+0.15**	6.41	**+0.22**	N.D.	N.D.
T2	2.11	−0.06	7.49	+0.01	6.38	−0.05	2.50/2.20	−0.02/−0.03
A3	8.30	+0.05	8.53	−0.13	6.50	+004	3.01/3.01	−0.07/−0.07
G4	11.52	**-0.28**	7.98	**-0.16**	6.22	+0.02	2.99/2.69	−0.11/−0.19
G5	11.18	**-0.24**	7.81	**+0.18**	6.10	−0.12	2.70/2.75	−0.15/−0.15
G6	10.70	**-0.50**	7.88	0.00	6.45	+0.01	2.82/2.73	0.00/+0.01
T7	1.80	00.0	7.55	0.00	6.15	−0.11	N.D./2.20	−0.16

Chemical shifts of selected d(TTAGGGT)_4_ protons were measured in ppm (δ) at 15°C and referenced from external standard (2,2-dimethyl-2-silapentane-5-sulfonate). *R=* 3.0 (PDZ-7/d(TTAGGGT)_4_), *Δδ* = *δ*_bound_ - *δ*_free_. N.D., not determined.

**Table 3:** Chemical shift values of PDZ-7 free and in the complex with d(TTAGGGT)_4_

PDZ-7	Free	Bound	*Δδ*
H-6	7.70	7.60	−0.10
H-5	6.89	6.69	−0.20
H-4	8.08	7.78	−0.30
H-1	7.72	7.25	−0.47
H-2	7.69	7.25	−0.44
H-3	7.69	7.25	−0.44
CH_2_N^+^	4.40/4.78	N.D.	N.D.
CH_2_N	3.50/3.00	N.D.	N.D.
CH_3_	2.90/2.99	2.60	−0.30/−0.39

Refer to Table 3. N.D., not determined.

## DISCUSSION

Preliminary *in vivo* efficacy studies on PDZ-7 indicated that this compound has a competitive advantage over doxorubicin in low dose treatment of human colon cancer xenografts. These optimistic results and future potential applications encouraged to study mechanism of this phenomenon. To this end, both DNA and actin cytoskeleton were identified as two targets for cytotoxic anthrapyridazone PDZ-7 in this work. Our mechanistic studies were based on previously published data [22], which suggested correlation between DNA affinity and activity of 23 anthrapyridazones. Interestingly, these two parameters have been shown to give poor correlations for anthraquinone analogues, including anthracyclines [47], and for four-ring heteroaromatic imidazoacridones [48]. Similarly to PDZ-7, these above-mentioned compounds [49] elicit cytotoxic activity through topoisomerase IIα inhibition. During the experiments related to Topo I inhibition the unique pattern of DNA migration in the presence of PDZ-7 was observed, similar to recently reported for isoindoloquinoxinaline derivatives [49]. This behavior was proposed to result from non-intercalative DNA binding. Structural studies using ^1^H and ^31^P NMR were conducted and confirmed external binding of PDZ-7 to the double helix. Our data suggest that nature of Topo I and Topo IIα inhibition by PDZ-7 is non-catalytic and could result from distortions of DNA structure, which prevent complex formation between topoisomerase and DNA [50]. Similar effect has been observed for other strong DNA-interacting compounds, which attenuate topoisomerase reaction cycle through non-catalytic mechanisms [50].

Despite low Topo IIα poisoning in cells, PDZ-7 rapidly induced double stranded DNA breaks as indicated by increased phosphorylation of histone H2AX and activation of DNA damage response (DDR). Therefore, it is conceivable that PDZ-7 induces DNA double-stranded breaks (DSB) through inhibition of strand passage reaction and stabilization of topoisomerase cleaved complex. However, it may additionally destabilize DNA structure or induce other forms of DNA lesions, such as single-strand breaks or covalent cross-links. These may ultimately convert to DSB [51], or create sensitive sites for topoisomerase cleavage [52]. Such interpretation is supported by the sensitivity of NER-deficient cells to PDZ-7 and DNA binding studies. Our results suggest that the endonuclease activity of XPG and XPF [53] is important for the removal of PDZ-7 induced DNA lesions, while helicase activity of XPD [54] and recognition of stalled RNAP by CSB is not [55]. Hypersensitivity of *BRCA2-* cells has been described for other DNA damaging compounds, including anthracyclines [35] or alkylators ecteinascidin 743 [37], temozolomide [56], all of which are clinically used drugs, or experimental acronycine derivative S23906-1 [36]. Works of Soares *et al.* [37] and Rocca *et al.* [36] are of particular relevance in this context, as they demonstrate sensitive phenotype of *BRCA2-* cells to DNA damage in comparison to cells deficient in other DNA repair proteins. Loss of BRCA2 protein compromises the repair of double-stranded DNA breaks (DSB) *via* Homologous Recombination (HR), the main DSB repair mechanism during S and G_2_/M phases [57]. Without functional HR even low concentration of PDZ-7 could be lethal due to accumulation unrepaired double strand breaks. Hypersensitivity of *BRCA2*- cells to PDZ-7 provide further evidence that DSB are the direct cause of anthrapyridazone cytotoxicity [36, 37].

Both PDZ-7 and BS-154 induced comparable level of γH2AX *foci* at equitoxic concentrations. It is also interesting to note that minor exchange of the side chain (N-methyl ethylamine in BS-154 to methyl group in PDZ-7) caused drastic change in anthrapyridazone cytotoxicity and tolerance in mice. Histone H2AX was phosphorylated at comparable levels from G_1_/S to G_2_/M phases of the cell cycle, with the only exception for G_1_ phase cells, which were less susceptible to the PDZ-7 induced DNA damage. Similarly, Darzynkiewicz *et al.* showed that exposure of A549 cells to mitoxantrone and etoposide, two Topo IIα poisons differing in DNA binding capacities, induced DDR throughout the cell cycle [28, 30] and DSB were not closely associated with the sites of DNA replication [27].

An interesting finding of our study is that PDZ-7 acts by more than one mechanism of action and can destabilize actin cytoskeleton independently of DNA damage. Actin cytoskeleton has long been proposed as a target for anti-cancer drugs, due to its implication in cancer invasion and metastasis [58]. However, direct actin polymerization inhibitors, such as cytochalasins or latrunculins are highly toxic [59]. In light of this, it is worth noting that PDZ-7 was well tolerated in mice, either due to sufficiently low actin depolymerisation activity or lower selectivity towards mouse actin. Further studies on this issue are required to fully understand this process.

The most effective small-molecule anti-cancer drugs elicit their activity on more than one pathway. With this regard, anthracyclines have been proposed to have about ten distinct modes of action [60, 61]. Recently they have been shown to attenuate DDR through displacement of histones (including H2AX) from damaged chromatin [11] and to interact with DNA G-quadruplexes [62]. On the other hand, cytoskeleton-destabilizing drugs inhibit trafficking of repair proteins, apart from their antimitotic activity [63]. Therefore it is often challenging to determine, which modes of action are truly important for the anticancer activity [60].

Therefore, here, we suggest that the inhibition of DNA synthesis and mitotic activity by PDZ-7 is, alike with anthracyclines, merely a consequence of DNA damage. In A549 cells, exposure time played decisive role with regard to dominant mechanism: at short exposure times PDZ-7 behaved as a typical DNA damaging agent, activating rapid DNA damage response and cell cycle arrest in G_2_ phase, supported by the observed pattern of growth arrest. However, longer exposures led to actin disassembly blocking cell cycle in G_1_ phase and in late S or tetraploid (bi-nucleate) G_1_. Additionally, we show that unusual cell cycle perturbation by PDZ-7 is associated with differential expression of cyclins A, B and E. Thus, it is predicted that in the *in vivo* setting, where drug concentration spikes, followed by rapid clearance from plasma, DNA damaging effect of PDZ-7 will be the most dominant.

Owing to its high activity and relatively low animal toxicity, PDZ-7 or related anthrapyridazones emerge as potential anticancer agents and encourage their further pre-clinical development. The exact nature of PDZ-7-induced DNA lesions is not fully understood and therefore requires further investigation. In particular, it is interesting to check whether PDZ-7 can induce DNA damage by interacting with enzymes other than DNA topoisomerases and if actin depolymerisation observed *in vitro* can be recorded in *in vivo* models.

## MATERIALS AND METHODS

### Drugs

Anthrapyridazones PDZ-7 and BS-154 were synthesized by Dr Malgorzata Wysocka (BLIRT S.A., Gdansk), as described [22]. Doxorubicin, etoposide, latrunculin A and SN-38 were purchased from Sigma-Aldrich (Poznan, Poland). All drugs were dissolved in DMSO to concentrations of 2 or 10 mM and stored at −20°C.

### *In vivo* experiments

*In vivo* toxicity studies were performed on 8-10 week old female BALB/c mice and *in vivo* therapeutic efficacy studies in athymic Foxn1nu mice. Mice were held in specific pathogen-free conditions and granted free access to food and water. To determine MTD, PDZ-7 was dissolved in 0.9% NaCl and single dose (10, 15, 30 or 50 mg of drug per kg body weight, 0.01 ml per 1 g body weight) was injected into BALB/c mouse tail vein (3 mice per dose). Body weight of mice was monitored every day for two weeks, after which time mice were sacrificed and subjected to anatomopathological analysis. In the initial *in vivo* efficacy study aimed to determine anticancer activity, 10 × 10^6^ LoVo/DX cells were subcutaneously transplanted into Foxn1nu mice (10 mice per condition). Tumors were allowed to grow to 70 mm^3^, after which mice were treated with three doses once a week (days 6, 13, 20) of 15 mg PDZ-7 (*ca.* 0.3 MTD; cumulative dose of 45 mg/kg) or 1 mg doxorubicin (*ca.* 0.3 MTD; cumulative dose of 3 mg/kg) per kg body weight. Tumor volume was determined every 3 days and mice were anesthetized with isoflurane and euthanized 24 days after xenotransplantation. All *in vivo* studies were performed according to Interdisciplinary Principles and Guidelines for the Use of Animals in Research, Marketing and Education issued by the New York Academy of Sciences’ Ad Hoc Committee on Animal Research and were approved by the First Local Committee for Experiments with the Use of Laboratory Animals, Wroclaw, Poland.

### Cell lines

A549 and HL-60 cells were from ATCC. Nalm-6 topoisomerase knock-out cells, originally obtained by Noritaka Adachi (Yokohama City University, Japan), were kindly provided by Caroline Austin and Ian Cowell (Newcastle University, UK). CHO-AA8, CHO-K1 and V79 cells, along with their DNA repair deficient variants, were provided by Malgorzata Zdzienicka (Leiden University, Netherlands). A549, HL-60, Nalm-6 and derived cells were cultured in RPMI-1640 medium (Corning 15-040-CV); CHO-K1, CHO-AA8 and all derived cells were cultured in F-10 Ham medium (Corning 10-070-CV). Media were supplemented with 10% FBS (Sigma–Aldrich F7524) and antibiotics: penicillin (60 μg/ml) and streptomycin (42.4 μg/ml). LoVo/DX cells were cultured in 1:1 (v/v) mixture of RPMI-1640 and Opti-MEM (both IIET, Wroclaw, Poland) supplemented with 5% FBS (F7524), 2 mM L-glutamine (G8540), 1mM sodium puryvate (P4562) (all Sigma-Aldrich, Poznan, Poland), 0.1 μg/ml doxorubicin (Medac GmbH, F130421A) and antibiotics: penicillin (60 μg/ml) and streptomycin (100 μg/ml). All cells were maintained at 37°C in humidified atmosphere of 5% CO_2_ and 95% air and routinely screened for *Mycoplasma* contamination.

### Actin polymerization assay

Experiment was carried out with Actin polymerization kit (Cytoskeleton #BK003) according to manufacturer's instructions. Briefly, 0.4 mg/ml pyrene-labelled rabbit muscle G-actin was suspended at in 0.2 mM CaCl_2_, 5 mM Tris-HCl, pH 8.0, allowed to depolymerize (1 hour, 4°C) and cleared by centrifugation (16,100 x g, 30 minutes, 4°C). Tested compounds diluted in DMSO (final concentration 1%) were added to G-actin solution and baseline fluorescence was determined (20 minutes, RT). Polymerization was initiated by the addition of 50 mM KCl, 2 mM MgCl_2_, 5 mM guanidine carbonate, 1 mM ATP, 10 mM Tris-HCl, pH 7.5 (final concentrations) and allowed to proceed for 1 hour. Data was gathered using Tecan Infinite M1000PRO in kinetic mode. Fluorescence was read using excitation wavelength set to 350 ± 20 nm and emission wavelength to 410 ± 10 nm every 30 seconds from the moment of compound addition until the signal reached plateau.

### Topoisomerase inhibition assays

Reaction mixture contained 50 ng pBR322 plasmid DNA (Thermo Scientific #SD0041), 40 mM Tris-HCl (pH 7.5), 120 mM KCl, 10 mM MgCl_2_, 1.1 mM EDTA, 10 mM β-mercaptoethanol, 100 μg/ml BSA and drug diluted in DMSO (final concentration 5%) was prepared and reaction was initiated by the addition of 5 μl (total reaction volume 10 μl) human recombinant Topo1 diluted in reaction buffer and allowed to proceed for 30 minutes at 37°C. Reactions were terminated by the addition of 5-times concentrated loading buffer (0.1% SDS, 5% sucrose, 2.5 mM EDTA 0.05% bromophenol blue in ddH_2_O, final concentrations). Samples were resolved in 1% agarose gel in TBE for 12 h at constant voltage of 1 V/cm and current not exceeding 20 mA. Gel was stained with ethidium bromide, destained in TBE and photographed under UV illumination.

For Topo IIα (Topogen TG2013), reaction mixture contained 200 ng kDNA, 50 mM Tris-HCl (pH 7.5), 150 mM NaCl, 10 mM MgCl_2_, 5 mM ATP, 0.5 mM DTT, 30 μg/ml BSA and reactions were terminated by the addition of 1% sarkosyl, 5% glycerol 0.05% bromophenol blue in ddH_2_O, final concentrations. Cleavage was performed with 200 ng pBR322 in same buffer and the reactions were stopped by addition of 0.3 mg/ml proteinase K in 0.35% SDS, 15 mM EDTA and topoisomerase digestion (90 minutes, 50°C), before adding loading buffer.

Topoisomerase inhibition in cells was determined using ICE assay as described [64]. In brief, cells were lysed in 1% sarkosyl in TE, DNA was sheared with 26G needle, separated on 1.5 g/ml CsCl_2_ cushion using SW55Ti rotor (Beckmann), (121,000 × *g*, 20 hours, 4°C) and spot-blotted on nitrocellulose membrane. Topoisomerases were detected with mouse anti-Topoisomerase I IgM (1:100, BD Biosciences 556597) and rat anti-IgM IgG_2a_, κ (1:200, BD Biosciences 553435) or anti-Topoisomerase IIα IgG_1_ (1:100, BD Biosciences 611327) and enhanced chemiluminescence (SuperWest Pico, Thermo Scientific).

### Drug sensitivity assay

A549, CHO-AA8, CHO-K1, V-79 and derived cell lines we seeded in 24-well plates at 4,000 cells/per well and allowed to attach overnight. Subsequently, drugs diluted in culture medium (final DMSO concentration 0.5%) were added and cells were incubated for 120 hours in the presence of drug. Nalm-6 cells were seeded at 50,000 cells/ml and drugs were added immediately. Cell survival was determined with MTT assay. Briefly, MTT diluted in PBS was added to a final concentration of 0.4 mg/ml and after 4 hours (37°C) medium was aspirated. Precipitated and dried formazan crystals were dissolved in DMSO and absorbance was read using 540 nm measurement filter and 650 nm reference.

### Flow cytometry

A549 cells were seeded in Petri dishes, allowed to attach overnight and treated as indicated. Drug-treated cells were harvested, fixed with 70% ethanol (overnight or longer, −20°C), rehydrated in PBS, stained with 20 μg/ml propidium iodide and 100 μg/ml RNase A in PBS (1 hour, RT) and analysed directly. To detect DNA synthesis, cells were labelled with 20 μM 5-bromo-2′-deoxyuridine (BrdU) for 1 hour before treatment end and fixed in 70% ethanol (overnight or longer, −20°C). Following brief rehydration with PBS (10 minutes on ice), DNA was denatured with 2 M HCl (30 minutes, RT) and suspension was neutralized with 0.1 M sodium tetraborate, pH 8.5 (10 minutes, RT). Cells were blocked with 1% BSA in PBS (15 minutes, RT), incubated with mouse anti-BrdU antibody (1:100, CalBioChem #NA61, 1 hour, 37°C), and incubated with anti-mouse FITC-conjugated donkey antibody (1:200, Jackson 715-095-151, 30 minutes, 37°C). To detect mitotic cells, ethanol-fixed cells were rehydrated with PBS (10 minutes on ice), blocked with 1% BSA in PBS and incubated with mouse anti-MPM2 antibody (1:100, DAKO M3514, 1 hour, 37°C), and anti-mouse FITC-conjugated donkey antibody (1:100, Jackson 715-095-151, 45 minutes, 37°C). To detect DNA damage, ethanol-fixed cells were rehydrated with PBS (10 minutes on ice), washed three times with 1% BSA in PBS and incubated with Alexa488-conjugated mouse anti-γH2AX antibody (1:40, BioLegend #613406, 1 hour, 37°C). For BrdU, MPM-2 and γH2AX, DNA was counterstained with 5 μg/μl propidium iodide and 100 μg/μl RNaseA in PBS (30 minutes, RT) and cells were processed by flow cytometry. 10,000 cells were scored for each assay.

### Immunofluorescence

A549 cells were seeded on coverslips, allowed to attach overnight and treated as indicated. Drug-treated cells were fixed and permeabilized in a single step with 50% acetone/methanol (2 minutes, RT), blocked with 0.5% BSA, 0.2% cold-water fish skin gelatin in PBS (30 minutes, RT), incubated with mouse anti-γH2AX antibody (1:200, Millipore 05-636, 60 minutes, 37°C), and anti-mouse Alexa-594 conjugated donkey antibody (1:100, Jackson Immuno Research 715-585-150, 30 minutes, 37°C). DNA was counterstained with 0.1 μg/ml DAPI (10 minutes, RT), coverslips were mounted in 25 mg/ml 1,4-diazabicyclo [2, 2, 2]octane (DABCO) in 50% glycerol in PBS and sealed with nail polish. Images were acquired under fluorescence microscope using identical settings for every sample. Fluorescence intensity of γH2AX was quantified as Median Fluorescence Intensity (MFI) over the nuclear area of each cell. Nuclear area was determined by applying automatic thresholding procedure on the images of DAPI-stained cells and γH2AX MFI was calculated inside the selected area. All operations were performed on greyscale 16-bit tiff images.

For α-tubulin, cells were fixed and permeabilized with methanol (20 minutes. −20°C), blocked with 1% BSA in PBS and incubated with mouse anti-α-tubulin antibody (1:300, Amersham N365, 60 minutes, 37°C) and anti-mouse DyLight-488 conjugated donkey antibody (1:100, Pierce SA5-10166, 60 minutes, 37°C). F-actin was stained with 0.5 μg/ml TRITC conjugated phalloidin (Sigma-Aldrich P1951, 20 minutes, RT) in PBS in cells fixed with 4% formaldehyde (10 minutes, 4°C) and permeabilized with 0.5% Triton X-100 (10 minutes, RT). DNA was stained as described above.

### Western blotting

Cells were lysed in RIPA buffer (5 mM EDTA, 0,1% (w/v) SDS, 1% (v/v) NP-40, 0,5% (w/v) sodium deoxycholate, 150 mM NaCl, 50 mM Tris pH 7.4) with an addition of the commercially available protease inhibitor cocktail (Roche) and phosphatase inhibitors: 50 mM NaF, 50 mM β-glycerophosphate and 1 mM Na_3_VO_4_ (30 minutes on ice). Lysates were cleared at 16,100 x *g* (4°C, 10 minutes) and protein concentration was measured with BCA assay (Thermo Scientific 23227). Equal amounts of protein were prepared in Laemmli Buffer and loaded on polyacrylamide (7.5, 10 or 12%) gels. SDS PAGE gels were resolved in Running Buffer (15 minutes at constant 100 V and 50 minutes at 200 V). Proteins were transferred on the pre-wet polyvinylidene fluoride (PVDF) membranes in Transfer Buffer (3 hours at constant current of 250 mA, 4°C). PVDF membranes were blocked in 5% BSA in TBST, and incubated (overnight, 4°C) with appropriate primary antibodies: anti-Cyclin A (1:200, Santa Cruz sc-596), anti-Cyclin B1 (1:100, Santa Cruz sc-245), anti-Cyclin D1 (1:1,000, Cell Signaling #2926), anti-Cyclin E (1:200, Santa Cruz sc-247), anti-cdc25c (1:200, Santa Cruz sc-327), anti-cdk (1:200, Santa Cruz sc-163), anti-Actin (1:600, Santa Cruz sc-1616), anti-α-tubulin (1:5,000, Amersham N356), anti-p-Nbs1 (1:250, Cell Signaling #3001), anti-Nbs1 (1:500, Cell Signaling #14956), anti-p-Mre11 (1:250, Cell Signaling #4859), anti-Mre11 (1:1,000, Cell Signaling #4847), anti-Rad50 (1:1,000, Cell Signaling #3427). Next, membranes were incubated with secondary anti-mouse, anti-rabbit and anti-goat antibodies (All at 1:10,000, Jackson ImmunoResearch 715-035-150, 711-035-152, 705-036-147) and X-ray films (Agfa) were developed using enhanced chemiluminescence (SuperWest Pico, Thermo Scientific).

### NMR

Oligonucleotides samples were prepared at a 0.25-0.40 mM concentration range, in H_2_O/D_2_O (9:1) containing 10 mM NaH_2_PO_4_/Na_2_HPO_4_ and 100 mM NaCl, pH 6.8 for the d(CGTACG)_2_ and d(AAGAATTCTT)_2_ oligonucleotides (Eurofins Genomics Italy, Milan). For the G-quadruplex d(TTAGGGT)_4_ studies buffer containing 25 mM KH_2_PO_4_, 150 mM KCl and 1 mM EDTA, pH 6.7 was used. The oligonucleotide samples were heated to 85°C for 1 minute and then cooled at room temperature overnight. Stock solutions of PDZ-7 were prepared in DMSO-d6.

The ^1^H spectra were acquired at variable temperature of 15°C and 25°C and were referenced to external DSS (2,2-dimethyl-2-silapentane-5-sulfonate sodium salt) set at 0.00 ppm. Chemical shifts (δ) were measured in ppm. ^31^P NMR spectra was recorded at 242.94 MHz and referenced at 1% H_3_PO_4_ (external reference). ^1^H NMR titrations were performed by adding increasing amounts of the drug to the oligonucleotide solution until *R*= [drug]/[oligonucleotide] = 2.0 or 4.0 was reached. Phase sensitive NOESY spectra were acquired in TPPI mode, with 2048 × 1024 complex FIDs and mixing times ranging from 200 ms to 400 ms. All spectra were transformed and weighted with a 90° shifted sine-bell squared function to 4K x 4K real data points. Pseudo two-dimensional DOSY [65] experiments were acquired using the pulse-program “stebpgp1s” and raw data were processed using the standard DOSY software present in the Bruker library (TOPSPIN v. 1.3). All NMR spectra were recorded on a Bruker AV600 spectrometer operating at a frequency of 600.10 MHz.

### Software and equipment

Microscopy images were captured with Olympus BX60 epifluorescent microscope coupled to XC50 CCD camera and analysed in Fiji [66]. Flow cytometry was performed using Guava EasyCyte 8HT (Merck) and analysed with Guava Soft 2.7 InCyte (Merck) or Flowing Software 2.5.1. Statistical analysis was performed with GraphPad Prism 5 and uniform significance levels were used through the entire manuscript: ^*^ *p*<0.05; ^**^ *p*<0.01; ^***^ *p*<0.001.

## Abbreviations

BrdU: 5-bromo-2′-deoxyuridine
BSA: bovine serum albumin
CHO: Chinese hamster ovary
DDR: DNA damage response
DOSY: diffusion-ordered NMR spectroscopy
DSB: double stranded break
DSS: 2,2-dimethyl-2-silapentane-5-sulfonate
H2AX: histone 2A.X
ICE: immune complex of enzyme
LanA: latrunculin A
MDR: multidrug resistance
MRN: Mre11-Rad50-Nbs1
MTT: (3-(4,5-dimethylthiazol-2-yl)-2,5-diphenyltetrazolium bromide
NMR: nuclear magnetic resonance
PBS: phosphate buffered saline
ROS: reactive oxygen species
Topo IIα(2β): topoisomerase IIα (IIβ)
